# Evidence for Transient Receptor Potential (TRP) Channel Contribution to Arthritis Pain and Pathogenesis

**DOI:** 10.3390/ph11040105

**Published:** 2018-10-15

**Authors:** Tabitha Galindo, Jose Reyna, Andy Weyer

**Affiliations:** 1 School of Physical Therapy and Athletic Training, Pacific University, Hillsboro, OR 97116, USA; tdgalindo@pacificu.edu (T.G.); reyn1741@pacificu.edu (J.R.); 2 Biological Sciences Department, City College of San Francisco, San Francisco, CA 94112, USA

**Keywords:** arthritis, osteoarthritis, rheumatoid arthritis, gout, TRPA1, TRPV1, TRP, transient receptor potential

## Abstract

Based on clinical and preclinical evidence, Transient Receptor Potential (TRP) channels have emerged as potential drug targets for the treatment of osteoarthritis, rheumatoid arthritis, and gout. This review summarizes the relevant data supporting a role for various TRP channels in arthritis pain and pathogenesis, as well as the current state of pharmacological efforts to ameliorate arthritis symptoms in patient populations.

## 1. Introduction

Arthritis is a debilitating disease resulting in pain and degradation of articular joint surfaces that affects millions of people around the world, but for which treatment options are often limited. Although there are many types of arthritis, each resulting from a slightly different pathology and presenting with slightly different clinical symptoms, the three most common forms are osteoarthritis, rheumatoid arthritis, and gout. The focus of this review will be on the intersection of these three kinds of arthritis with transient receptor potential (TRP) channels, a family of proteins whose contribution to the pain and pathophysiology of arthritis has been increasingly studied over the past decade and whose members represent possible drug targets for the amelioration of arthritic pain and joint degeneration.

## 2. Osteoarthritis, Rheumatoid Arthritis, and Gout: Pathophysiology and Current Treatments

Osteoarthritis (OA) is the most common form of arthritis in adults, affecting more than 13% of the world’s population. It is characterized by progressive deterioration and loss of articular cartilage, which leads to substantial modifications of the various tissues within and surrounding the joint capsule [1,2,3,4,5,6]. This deterioration seems to be triggered by inflammatory mediators like interleukins and tumor necrosis factor alpha (TNFα), as well as oxidative stress, but the ultimate etiology is likely a complex interplay between environmental factors, genetics, and alterations in load-bearing by articular surfaces [2,7,8]. Conservative management of OA is focused primarily on pain alleviation via non-steroidal anti-inflammatory drugs (NSAIDs), corticosteroid injections, physical therapy, and the use of assistive devices. These interventions typically result in moderate improvements in pain and function over the short term [9,10,11,12], but none of these treatments is ultimately able to halt disease progression. Additionally, long-term use of NSAIDs is associated with the development of gastrointestinal complications and increased risk of cardiovascular events, which is especially concerning given that patients with OA are often older and have multiple co-morbidities. Furthermore, corticosteroid injections at higher dosages or longer durations are known to cause additional damage to articular cartilage [11,13,14,15]. Total joint arthroplasty has good outcomes and serves as a permanent solution for individuals suffering from OA. However, it is not appropriate for all individuals suffering from this condition, including individuals with multiple co-morbidities, individuals who are morbidly obese, younger individuals (who would likely require a revision later in life), and those with limited access to expensive healthcare treatments. Thus, when considering the number of patients whose symptoms are unresolved given current treatment options, it is clear that novel therapies targeting both pain and OA progression are needed.

Gout, another form of arthritis, is characterized by the accumulation of monosodium urate (MSU) crystals both within and around a joint, and has been estimated to affect almost four percent of the population in the United States [5,16,17]. In contrast to OA and rheumatoid arthritis (RA), individuals suffering from gout endure intermittent acute episodes that last 7–10 days prior to spontaneous resolution. Episodes are most common in the foot and ankle, and patients typically complain of debilitating pain, edema, tenderness, warmth, and loss of joint mobility [18,19,20,21,22]. Current first-line therapies to treat acute gout flares include glucocorticoids, NSAIDs, and colchicine, all of which may aid in stemming the inflammatory component of gout. However, these drugs are largely non-specific and can cause a variety of adverse events, especially in older patients or those with co-morbidities [23]. Furthermore, these drugs provide significant reductions in pain and increases in function in only about 50% of patients to whom they are prescribed [24,25]. For individuals suffering from repeated gout attacks, xanthine oxidase inhibitors such as febuxostat and allopurinol, as well as uricases like pegloticase, are utilized to lower uric acid levels throughout the body [26,27]. However, these drugs can lead to increased flare-ups and have numerous side effects such as diarrhea, nausea, dizziness, headaches, liver and kidney abnormalities, and musculoskeletal issues. Additionally, there appear to be increased rates of cardiovascular events in individuals taking febuxostat [28]. Finally, for patients not responding to other treatments, numerous studies have explored whether inhibition of interleukin-1 (IL-1), one of the prime players in the pathophysiology of gout, is an effective pharmacologic strategy. While inhibition of IL-1 has been shown in studies to reduce pain and improve function in patients suffering from gout [22,29,30,31], the Food and Drug Administration (FDA) has denied approval of these compounds for the treatment of gout due to long-term safety and efficacy concerns [32,33]. Thus, similar to OA, the identification of novel therapeutics for the treatment of gout is critical.

In contrast to OA and gout, RA is an autoimmune disease that affects the synovium, articular cartilage, and bone within a joint, typically in a symmetrical distribution [34,35,36]. Affected joints are infiltrated with mononuclear cells such as T cells and B cells, as well as macrophages, and patients typically complain of joint pain, swelling, and stiffness in the metacarpophalangeal joints and proximal interphalangeal joints of the hand, the wrist, the foot, or the upper limb [37,38,39,40]. First-line treatment for RA consists of disease-modifying anti-rheumatic drugs (DMARDs) like methotrexate that work to suppress the immune system, and these are often used in combination with NSAIDs to provide faster relief [41,42,43]. However, because DMARDs suppress the immune system, they may lead to an increased risk of infection, and recent studies have found that concomitant use of low-dose methotrexate along with NSAIDs increases the risk of serious adverse events such as renal failure and cytopenia [40,44,45,46]. Furthermore, these drugs are unable to provide immediate relief in the event of a flare-up.

Collectively, while current treatments for arthritis provide efficacy for some patients, it is also clear that arthritic symptoms are refractory to pharmacological treatment for many others, and that an additional cohort of patients consists of individuals who are not appropriate candidates for current pharmaceuticals. Thus, the search for additional drug targets must continue, and, as will be detailed in the following sections, members of the TRP family of ion channels present an excellent starting point for this endeavor. Of note, because TRP channels have been largely studied in the context of animal models of arthritis that may or may not specifically replicate the disease processes found in humans, this review will examine the evidence for TRP channel involvement in the pathogenesis of arthritis models as a whole, rather than breaking down the evidence for each TRP channel in each animal model. 

## 3. Transient Receptor Potential Vanilloid 1 (TRPV1)

TRPV1 is the most well-studied of the TRP channel family members, and this also holds true in the context of arthritis research. TRPV1 has been consistently implicated for playing a role in animal models of OA, RA, and gout, and has also been linked to these diseases in humans. 

TRPV1 is best known for its function in nociception, especially in response to heat and inflammatory compounds, and the literature suggests that it serves a similar function in joint afferents. TRPV1^−/−^ animals display reduced pain following Complete Freund’s Adjuvant (CFA)-mediated arthritis induction compared to TRPV1^+/+^ animals as judged by weight-bearing ratio between the affected and unaffected legs [47]. Injection of the TRPV1 antagonists A-889425 and JNJ-17203212 systemically reduced pain behaviors in the monoiodoacetate (MIA) model of arthritis pain, and also reduced the firing of peripheral A- and C-fiber joint afferents, as well as the firing of nociceptive and wide dynamic range neurons in the dorsal horn of the spinal cord in response to peripheral stimuli in these animals [48,49]. Likewise, injection of the TRPV1 antagonist capsezapine into the inflamed temporo-manibular joint of rats resulted in significantly reduced pain behaviors compared to controls [50], and injection of the TRPV1 antagonists SB366791, AMG9810, and ABT-116 in murine models of gouty arthritis also significantly reduced pain behaviors [51,52,53]. Additionally, a number of studies have demonstrated that treatment with TRPV1 agonists such as capsaicin or resiniferatoxin prior to arthritis induction leads to reduced pain and joint damage in arthritis models [51,52,54,55,56,57,58,59,60,61]. It has been hypothesized that application of these agonists at sufficient levels causes a long-lasting desensitization or degradation of TRPV1-expressing nociceptive afferents [62], which, in turn, leads to reduced pain sensation and release of neuropeptides responsible for potentiating the inflammatory response and overall joint pathology [57,63,64,65]. Indeed, this TRPV1 agonist-induced analgesia has been observed in humans, as topical capsaicin application has long been used by some patients as a treatment for arthritis pain, and a review on this topic identified a number-needed-to-treat of only 3.3 for a 50% reduction in pain [66]. Genetic data also implicates TRPV1 as a major player in arthritis pain, as the Ile585Val genetic variant within the coding region of the TRPV1 gene is correlated with reduced symptomatic pain complaints in patients suffering from OA [67].

However, TRPV1’s role in arthritis-related pain sensation is not without some controversy, as a study by Ängeby Möller et al. found no reduction in guarding behavior in an adjuvant-arthritis model in response to oral administration of the TRPV1 antagonist AZD1386 [68], and a study by Okun et al. found no reduction in pain behaviors as judged by a conditioned place preference assay in response to antagonism of TRPV1 with AMG9810 [69]. And while another study observed that resiniferatoxin pre-treatment reduced pain behaviors in a serum-transfer model of arthritis that closely mimics the disease process of RA, it also found that this desensitization of TRPV1-containing afferents had no effect on the amount of edema and destruction observed in the affected joints [56].

In addition to mediating arthritic pain, a wide body of evidence suggests that TRPV1 may also play a role in facilitating the joint destruction and edema that is characteristic of arthritis. Indeed, multiple studies have demonstrated that either pharmacological inhibition or genetic deletion of TRPV1 results in reduced joint swelling [47,51,52,70,71]. Another study observed significantly reduced destruction of articular tissues in TRPV1 knockout animals in an adjuvant arthritis model [72], although an earlier study utilizing the same model found that wild-type and knockout animals had similar levels of joint destruction [47]. These findings are likely the result of TRPV1’s recognized role in neurogenic inflammation, whereby its activation on sensory nerve terminals by inflammatory compounds leads to concomitant neuropeptide release from these afferents that subsequently potentiates the inflammation and vasodilation/vascular leakage. Indeed, a study by Fernihough et al. observed increased calcitonin gene-related peptide (CGRP) and TRPV1 expression and co-localization in the MIA model of OA [73].

Importantly, while TRPV1 has most frequently been studied within the peripheral nervous system based on its role in heat and pain sensation, much data has also identified the presence of TRPV1 in other cell types found in joints, particularly in chondrocytes and fibroblast-like synoviocytes. Gavenis et al. first identified the expression of the TRPV1 gene in chondrocytes [74], and subsequent studies of human articular cartilage observed that exposure of chondrocytes to the pro-inflammatory molecules interleukin-1 beta (IL-1β) and TNFα causes the chondrocytes to increase their gene expression of TRPV1 [67]. However, this study found no differences in basal levels of TRPV1 gene expression when comparing chondrocytes obtained from patients with OA to those obtained from healthy controls [67]. Whether and how these alterations in TRPV1 levels affect the function of chondrocytes and the overall pathogenesis of arthritis is still to be determined, although results from a recent study of chondrocytes obtained from mouse and chicken embryos hints that TRPV1 activity may affect chondrocyte proliferation and extracellular matrix production [75].

Synovial fibroblasts are another non-neuronal cell type that plays an integral role in joint health. These cells are responsible for generating components of the synovial fluid, including hyaluronic acid (HA). HA is known to increase the viscosity of synovial fluid, which aids in lubricating and cushioning the joint surfaces during movement, and also has chondroprotective effects based on its interactions with a variety of inflammatory molecules [76]. In osteoarthritic joints, however, there is degradation and dilution of HA, which leads to reduced viscosity of the synovial fluid and increased levels of inflammatory molecules, ultimately resulting in increased transmission of joint forces and degradation of articular cartilage [76]. Clinically, injections of exogenous HA into arthritic joints have been controversial, although a recent systematic review of meta-analyses concluded that this is an efficacious treatment for reducing pain and increasing function in patients suffering from OA [77]. Importantly, a study by Caires et al. may shed light on the effectiveness of HA in reducing pain, as data from these experiments indicated that HA may actually act to stabilize the closed state of TRPV1, leading to reduced pain sensation in the presence of nociceptive stimuli [77]. 

TRPV1 may not only serve as a target of HA produced by synoviocytes, but may also influence the release of HA from synoviocytes. Previous studies have identified both expression of the TRPV1 gene and functional TRPV1 channels in synovial fibroblast cell lines and synovial fibroblasts obtained from patients, indicating that activation of TRPV1 may affect the release of HA and other molecules from fibroblast-like synoviocytes via intracellular signaling pathways [78,79,80]. Indeed, activation of TRPV1 in synovial fibroblast cell lines and synovial fibroblasts from patients with OA or RA has been shown to lead to increased production of the inflammatory molecules prostaglandin E2, interleukin-6, and interleukin-8 [50,80,81]. These molecules may trigger much of the tissue degradation that is characteristic of arthritis and may subsequently initiate long-term changes in other cells of the joint space. For instance, direct contact between healthy sensory neurons and synovial fibroblasts obtained from arthritic animals has been shown to upregulate TRPV1 expression in the sensory neurons [82]. Furthermore, synoviocytes themselves undergo a noted hyperplasia upon stimulation by interleukins that contributes to pannus formation and mononuclear cell invasion, critical events that ultimately lead to cartilage and subchondral bone destruction [83,84]. Interestingly, evidence from synovial fibroblast cell lines suggests that TRPV1 in synovial fibroblasts does not become functionally active until slight acidification of the extracellular environment occurs, a finding that aligns with data indicating that inflamed joints typically have a pH below 7.0 [78,85,86]. This finding is not without some controversy, however, as another study by Hu and colleagues observed reduced capsaicin responses in cultured synovial fibroblasts obtained from animals with collagen-induced arthritis following reductions in pH [87].

As a result of the hypoxic, pro-inflammatory environment present in both OA and RA, reactive oxygen species (ROS) are generated [88,89]. This ROS generation has been shown to be due in part to TRPV1 activation on synovial fibroblasts [79,90], and the ROS are then able to participate in a positive feedback loop because TRPV1 itself is activated by ROS in synoviocytes and exhibits increased expression following ROS-mediated activation [90]. 

**Figure 1:**
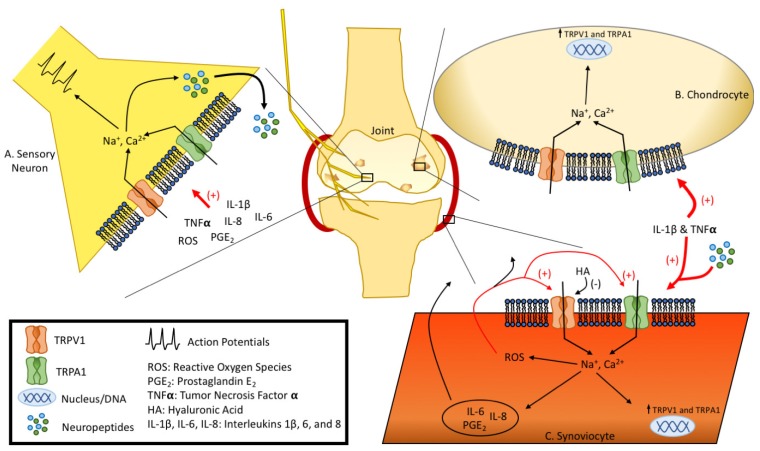
Summary of TRPV1 and TRPA1 Channel Involvement in Arthritic Joint Degradation. (**A**) Activation of TRPV1 and TRPA1 on sensory neurons may lead to neuropeptide release and pain sensation; (**B**) Activation of TRPV1 and TRPA1 on chondrocytes may lead to increased TRPV1 and TRPA1 gene expression; (**C**) Activation of TRPV1 and TRPA1 on synoviocytes may lead to production of ROS, release of inflammatory factors, and increased TRPV1 and TRPA1 gene expression.

Ultimately, TRPV1 clearly plays a central role in the pathology of OA, RA, and gout, with a variety of studies indicating that the activation of TRPV1 leads to the release of inflammatory compounds that initiate and potentiate the disease process, while also serving as a target of many of these same molecules (Figure 1). Thus, the activation of TRPV1 largely appears to serve as part of a positive feedback mechanism that works to enhance the disease process and pain of arthritis. Furthermore, because TRPV1 is located on sensory nerve endings, synovial fibroblasts, and chondrocytes, there is high potential for signaling between these different cell types to further potentiate the pathology. For these reasons TRPV1 is an excellent pharmaceutical target, as interfering with its activity may alter the functioning of multiple signaling mechanisms to reduce both pain and tissue degradation.

## 4. Transient Receptor Potential Ankyrin 1 (TRPA1)

While TRPV1 has been more frequently studied in relation to arthritis pathogenesis, there is ample evidence of a role for TRPA1 in models of OA, RA, and gout as well. 

Numerous studies have found that antagonism of TRPA1 normalizes the mechanical hypersensitivity observed after arthritis induction. In the CFA and MIA models of arthritis of the knee, the TRPA1 antagonists HC-030031 and AP-18 relieved behavioral sensitization via elevation of mechanical withdrawal thresholds and normalization of weight-bearing between the arthritic and healthy hind-legs [91,92]. Similarly, in the monosodium urate (MSU) model of gouty arthritis, HC-030031 was found to reverse the induced mechanical sensitization [60,61]. Furthermore, genetic deletion of TRPA1 also normalized mechanical withdrawal thresholds and weight-bearing in models of arthritis and gout [60,61,91,92,93,94,95]. Interestingly, Garrison and Stucky also examined the contribution of TRPA1 to adjuvant-induced arthritis, but within the context of aging by using geriatric (>18-month-old) mice. Their findings suggest that TRPA1 is critical for mechanical sensitization during the acute, but not chronic, stage of inflammation in young animals, while its genetic deletion prevents mechanical pain during both acute and chronic arthritis in aged animals [96]. In contrast, another group failed to observe pain relief from MIA-induced knee arthritis in response to systemic or intra-articular HC-030031 application as judged by a conditioned place preference assay [69]. Cumulatively, however, the evidence supporting a central role for TRPA1 in the pain component of various types of arthritis is quite strong.

The mechanism by which TRPA1 is activated during arthritic degeneration appears to be a result of inflammatory factors such as TNF-α and IL-1β. Indeed, Hatano and colleagues observed expression of TRPA1 within cultured synovial fibroblasts only after they were exposed to TNFα or IL-1β [97]. Similarly, recent studies have found that exposure of human chondrocytes obtained from individuals with OA to IL-1β increased activity of the pro-inflammatory COX-2 pathway and expression of TRPA1 mRNA, an effect that was partially blocked upon exposure to the TRPA1 antagonist HC-030031 [94,98]. Furthermore, this effect was completely ameliorated in the MIA model of OA in TRPA1 knockout mice [94]. Considering the efficacy of anti-TNFα agents in the treatment of RA [99], the fact that TRPA1 appears to be downstream of TNFα in the pathology of arthritis positions it as a potentially important drug target. In addition to TNFα and IL-1β, multiple reports have also shown that ROS are elevated in models of arthritis, which are known to be activators of TRPA1 [100,101]. Indeed, blockade of TRPA1 reduces pain behaviors in response to injection of H_2_O_2_ in the MSU model of gout [60,61,95].

While the evidence for TRPA1’s involvement in pain sensation and activation by inflammatory factors during arthritic conditions is strong, there have been conflicting findings regarding its contribution to joint edema and tissue degradation. Some groups have found no effect of either pharmacological antagonism of TRPA1 with AP-18 or genetic knockout of TRPA1 on either joint edema or degradation [92,96]. In contrast, others have noted a significant reduction in edema and joint degradation when TRPA1 was either blocked or genetically deleted [60,61,93,94,95]. In support of a role for TRPA1 in edema promotion, Fernandes and colleagues observed increases in blood flow to the knee in an adjuvant-induced arthritis model, which was prevented by antagonism or genetic knockout of TRPA1 [91]. Although it is unclear what underlies these opposing findings, part of the mechanism through which TRPA1 may contribute to edema and joint destruction during arthritis is by triggering the release of inflammatory compounds via neurogenic inflammation. In the MSU model of gout, data indicate that secretion of neuropeptides and cytokines like CGRP, IL-1β, and IL-6 is reduced in the presence of HC-030031 and upon genetic deletion of TRPA1 [60,61,95]. Furthermore, these same studies and an additional report revealed a reduction in neutrophil infiltration into the joint following antagonism of TRPA1 or when TRPA1 is genetically deleted [60,61,93,95]. This reduced neutrophil infiltration is a critical finding, as these cells contribute to the edema and joint degradation observed in arthritis, especially RA, via further release of cytokines and proteases, and also contribute to ROS generation [102].

Finally, it is important to note that multiple studies have also observed alterations in the gene expression of TRPA1 in various tissues under arthritic conditions. Fernandes et al. observed increased gene expression of TRPA1 in articular cartilage and decreased gene expression of TRPA1 in synovial fibroblasts in an adjuvant-induced arthritis model when animals were housed at cold temperatures, but found no changes in the expression of TRPA1 in sensory neurons under these conditions [91]. These findings are particularly interesting given TRPA1’s controversial role in cold sensation and the known exacerbation of arthritic symptoms in response to cold conditions in humans [103,104,105,106]. Additional evidence at room temperature in an MSU model of gouty arthritis also identified an increase in TRPA1 protein expression in both joint tissues and the skin [60,61]. 

Collectively, these findings demonstrate that TRPA1 is an integral component within the pathogenesis of multiple types of arthritis (Figure 1). The findings concerning TRPA1’s involvement in gout are particularly strong and consistent, and future pharmaceutical development focusing on this channel are likely warranted. 

## 5. The Role of Other TRP Channels in Arthritis

Although the TRPA1 and TRPV1 channels have received the greatest attention for their role in arthritis-related models, several other TRP channels have also been studied in this context. Indeed, limited evidence suggests the involvement of transient receptor potential canonical 5 (TRPC5), transient receptor potential melastatin 3 (TRPM3), transient receptor potential melastatin 7 (TRPM7), transient receptor potential melastatin 8 (TRPM8), transient receptor potential vanilloid 2 (TRPV2), and transient receptor potential vanilloid 4 (TRPV4) in the pathogenesis of arthritis in animal models.

TRPC5 is expressed in multiple tissues and is known to associate with its family member transient receptor potential canonical 1 (TRPC1) to form heteromeric cation channels [107]. Recent evidence suggests that both TRPC5 and the TRPC5-C1 heteromultimers are functionally expressed in fibroblast-like synoviocytes obtained from patients with RA [108]. Interestingly, these channels are activated by extracellular reduced thioredoxin, which is present in significantly elevated extracellular concentrations in patients with RA [108,109]. Furthermore, suppression of TRPC5, through genetic deletion or pharmacological antagonism, results in amplified joint inflammation and hyperalgesia in human and murine models of RA [110]. Thus, activation of TRPC5 may be associated with an endogenous anti-inflammatory pathway that seeks to limit disease progression [108,110]. 

TRPM3 is expressed primarily in the kidney, but occurs in a broad distribution of other tissues as well [111]. It is a known steroid hormone sensor and is highly permeable to Ca^2+^ [107,111]. A recent study confirmed the expression of TRPM3 in synoviocytes obtained from humans suffering from RA, and activation of these channels resulted in a sustained Ca^2+^ response. Importantly, stimulation of TRPM3 via extracellular application of the steroid pregnenolone sulphate inhibited HA secretion by synoviocytes [112]. This is particularly interesting because some studies have implicated excessive secretion of HA as contributing to RA, while others indicate that HA serves an anti-inflammatory role in RA, much like its disease-mitigating role in OA [112,113,114,115,116]. This discord is perhaps explained by the findings of Roth et al., which indicate that HA is anti-inflammatory during early chronic RA, but transitions to a pro-inflammatory effect during late chronic RA in an animal model [117]. Ultimately, further studies are needed to determine whether either agonism or antagonism of TRPM3 is a viable strategy for altering HA levels and disease course in RA.

TRPM7 is ubiquitously expressed, permeable to multiple cations, and involved in a variety of roles, including cell cycle control [107,111]. TRPM7 activation is a key step in Ca^2+^ mobilization associated with CD147-enhanced invasiveness of human neutrophils in RA [118]. In the CFA model of arthritis in rats, TRPM7 expression is upregulated in fibroblast-like synoviocytes. Subsequent suppression of these TRPM7 channels yielded increased endoplasmic reticulum stress and synoviocyte apoptosis [119]. Thus, antagonism of TRPM7 ought to be further investigated in neutrophils and synoviocytes, cells which are both highly implicated in the pathology of RA [118,119].

TRPM8 plays a known role in pain and temperature sensation within sensory neurons and is found in prostate and certain cancer cells [107]. Cooling stimuli (cold temperature, chemical agonism) activate TRPM8 [107,120]. Zhu and colleagues demonstrated TRPM8-mediated Ca^2+^ entry plays an essential role in menthol-evoked apoptosis of synoviocytes obtained from animals with collagen-induced arthritis [120]. Because hyperplasia and secretion of pro-inflammatory compounds are two known roles of synoviocytes in arthritis, further investigation of intra-articular application of menthol or other TRPM8 agonists may reveal a mechanism leading to reduced pathology of joint tissues under arthritic conditions. 

TRPV2 is present in a wide variety of tissues with the signaling pathways for channel activation varying according to tissue type [121]. Interestingly, although TRPV2 gene and functional expression is increased in synoviocytes from patients with RA, TRPV2 agonists reduce the in vitro invasiveness of these synoviocytes, as measured by a 24-h invasion assay. Importantly, this in vitro invasiveness is known to correlate with histological and radiographic damage caused by the destructive RA pannus [122]. Thus, TRPV2 agonism should be further studied to confirm these findings and the therapeutic outcomes of decreased histological damage, inflammation, and angiogenesis suggested by this data [122].

TRPV4 is commonly expressed in multiple cell types and activated by a wide number of stimuli including multiple endogenous substances, hypotonic cell swelling, shear stress, and heat [107,111,123]. Interleukin-17 (IL-17), which has a known role in the pathogenesis of psoriatic arthritis and RA, upregulates the expression of TRPV4 receptors and intensifies mechanical hyperalgesia [124,125]. In synoviocytes obtained from rats in a collagen-induced arthritis model, TRPV4 is a key channel in controlling hypotonicity-induced Ca^2+^ entry and subsequent proliferation of synoviocytes, thus further aggravating the disease state [126]. However, in another study TRPV4^−/−^ mice were shown to present with rapid, severe development of joint degeneration not observed in TRPV4^+/+^ mice, suggesting that TRPV4 instead plays a protective role in the development of OA [127]. Therefore, similar to the current literature concerning TRPM3’s role in arthritis, further studies are warranted to determine whether TRPV4 serves a protective or pro-inflammatory role in arthritis development.

In summary, the current data suggests that *agonism* of TRPC5, TRPM3, TRPM8, and TRPV2 may help ameliorate or prevent arthritis, while *antagonism* of TRPM7 and TRPV4 may have a similar effect. Although the body of evidence regarding these channels in arthritis is somewhat limited, the available data does indicate that these targets are worthy of future study as it pertains to drugs for arthritis pain and pathogenesis. 

## 6. Pharmaceuticals Targeting TRP Channels for Arthritis Pain and Disease Progression 

A number of TRPV1 inhibitors have been developed and tested in clinical trials to treat acute and chronic pain, but to date none of these compounds has been approved for clinical usage due to lack of efficacy, elevations in core body temperature, and dangerous elevations in heat pain threshold [128,129,130,131]. At least three different TRPV1 inhibitors have proceeded to clinical trials specifically for patients suffering from OA. GRC 6211 was a TRPV1 antagonist developed by Glenmark Pharmaceuticals in conjunction with Eli Lilly that showed preclinical promise in the treatment of OA and bladder cystitis, but was ultimately halted during Phase II trials for undisclosed reasons [132,133]. AZD1386, a TRPV1 antagonist developed by AstraZeneca, was given to patients suffering from OA that was refractory to NSAIDs and acetaminophen. Unfortunately, despite pre-clinical efficacy, no differences were noted in pain scores on the Western Ontario and McMaster Universities Arthritis Index (WOMAC) or the numerical rating scale (NRS) for patients with OA given the drug versus those randomized to the placebo [134]. Post-hoc data analysis also identified a subset of patients allocated to the higher dosage group that exhibited increased alanine and aspartate aminotransferase levels, which is indicative of potential liver damage, after four weeks. Ultimately, AstraZeneca decided to abandon further trials for OA with AZD1386 based on these results [134].

Recently, the NEOMED Institute published data regarding a novel TRPV1 antagonist that only targets the capsaicin binding site on TRPV1, while not impacting its proton-binding or temperature activation sites in hopes of avoiding the pitfalls that plagued earlier attempts at inhibiting TRPV1. This drug, termed NEO6860, passed phase I trials without any negative effects on core body temperature, heat pain tolerance, or heat pain threshold [135]. In follow-up proof-of-concept Phase II trials in patients diagnosed with OA, NEO6860 generated some analgesia compared to baseline, but was not as effective in reducing arthritis pain as naproxen, an NSAID, compared to the placebo [136]. Given the analgesia that was generated, the research team has decided to continue testing this compound using different dosages and in combination with naproxen. 

While one pharmaceutical strategy emphasizes the inhibition of TRPV1, a completely different approach taken by the FDA-approved drug zucapsaicin (marketed as Civamide) and an in-development drug known as CNTX-4975 is to activate TRPV1 for the reduction of pain. Zucapsaicin is the *cis* isomer of capsaicin and is applied topically as a cream to cause desensitization of nociceptors in much the same way that capsaicin and resiniferatoxin pre-treatment are used to prevent arthritic pain in animal models [51,52,54,55,56,57,58,59,60,61]. By applying the drug topically, systemic absorption does not occur, thus avoiding the potential for off-target effects. While capsaicin creams have long been used as treatment for OA with varying levels of efficacy [66,137], the specific purported advantages of zucapsaicin over capsaicin are that it creates less local skin irritation upon application and greater efficacy in pre-clinical studies [138]. In a double-blind, randomized, controlled trial, 0.075% zucapsaicin cream was applied three times daily for three months in patients with OA for whom NSAIDs lacked efficacy. The study results indicated that 0.075% zucapsaicin created a statistically significant improvement in pain and function on the WOMAC [139], and these results were also clinically significant, having surpassed the identified minimal clinically important difference (MCID) for this outcome measure [140].

CNTX-4975, on the other hand, is a synthetic and ultrapure form of the *trans* isomer of capsaicin that is injected at 1 mg directly into the knee joint. Although peer-reviewed manuscripts concerning the effect of CNTX-4975 on OA have not yet been published, conference presentations and press releases by Centrexion Therapeutics have reported a statistically significant reduction in pain and increase in function in patients 6 months after a single injection of the compound [141,142]. Because of its favorable safety profile, the FDA supported fast-track status for this drug, which may lead to its approval within the next few years [143]. Rather than desensitizing nociceptive afferents like zucapsaicin, CNTX-4975 is thought to work via degeneration of nociceptive joint afferents via strong activation of TRPV1, resulting in its much longer duration of action. 

Interestingly, it has also been reported that the gold compound auranofin, a disease-modifying antirheumatic drug, is a potent agonist of TRPA1 [144]. Although auranofin’s clinical effects in suppressing the inflammatory component of RA are attributed to its inhibition of redox enzymes like thioredoxin reductase [145], it is intriguing that this compound also activates TRPA1 (at least in vitro), an effect that would theoretically increase pain and joint degradation given the evidence for its role in arthritis pathogenesis. One possibility is that, much like the desensitization of small diameter joint afferents by TRPV1 agonists such as zucapsaicin, auranofin works to desensitize TRPA1-expressing C fibers (many of which co-express TRPV1) innervating joint tissues. Desensitization of peripheral afferents as a result of TRPA1 activation has been observed in both clinical studies and animal models, resulting in an ultimate decrease in the release of neuropeptides such as CGRP that promote disease progression and pain [146,147]. Based on a literature search, follow-up studies on auranofin and its effects on TRPA1 in in vivo systems have not yet been performed, and no additional manuscripts examining TRPA1 agonists for the treatment of arthritis could be identified. However, given the success of TRPV1 agonists as topical treatments for OA, further studies on the effects of TRPA1 agonists for RA, OA, or gout are likely warranted. 

One additional avenue for future pharmaceutical research concerns targeting both TRPA1 and TRPV1 simultaneously. In a preclinical study using the MSU model of gout, Trevisan and colleagues demonstrated that the pain and swelling caused by H_2_O_2_ injection was unaffected by low doses of either HC-030031 (a TRPA1 antagonist) or SB-366791 (a TRPV1 antagonist) alone, but injection of low doses of these two antagonists simultaneously significantly reduced pain and swelling [60]. Despite the problems with first-generation TRPV1 antagonists due to off-target effects on core body temperature and heat pain threshold, these results hint at the possibility that application of TRPV1 antagonists at sub-therapeutic dosages in conjunction with novel TRPA1 antagonists could prevent adverse events while still providing clinical efficacy in humans. 

Finally, despite the strong evidence for TRP channel involvement in arthritis pain and pathogenesis, as novel TRP channel inhibitors are developed, additional consideration and attention must be given to the role that TRP channels play in normal joint homeostasis. For instance, in addition to pain reduction, inhibition of these channels could potentially lead to unanticipated effects on joint health by interfering with normal joint processes that then results in disease worsening. Indeed, there is precedence for this, as a small subset of patients with OA randomized to the anti-NGF treatment in clinical trials displayed rapidly progressive osteonecrosis in one or more joints [148,149]. Further analysis suggested that a potential contributor to the development of this adverse event was simultaneous treatment with NSAIDs [150]. Thus, attention must also be given to the interaction between TRP channel inhibitors and common over-the-counter treatments for arthritis. 

## 7. Conclusions

Both preclinical and clinical data strongly support a role for various TRP channels, especially TRPA1 and TRPV1, in arthritis pain and pathogenesis. Both TRPA1 and TRPV1 seem to mediate their effects on nociception as a result of their localization on joint afferents, while also serving as central mediators of disease progression secondary to their activation in synoviocytes and chondrocytes of affected joints. Various pharmaceuticals have been developed targeting TRPV1, but few of these compounds have actually proceeded to market. Further efforts to target TRPV1 and TRPA1 are certainly warranted based on the literature, and additional studies examining other TRP channels may lead to further prospects for ameliorating arthritic pain.
